# SGLT2 Inhibition in Heart Failure with Preserved Ejection Fraction — The New Frontier

**DOI:** 10.31083/j.rcm2401001

**Published:** 2023-01-03

**Authors:** Inês Aguiar-Neves, Diogo Santos-Ferreira, Ricardo Fontes-Carvalho

**Affiliations:** ^1^Cardiology Department, Centro Hospitalar de Vila Nova de Gaia/Espinho, 4434-502 Vila Nova de Gaia, Portugal; ^2^Cardiovascular R&D Centre – UnIC@RISE, Department of Surgery and Physiology, Faculty of Medicine of the University of Porto, 4200-450 Porto, Portugal

**Keywords:** SGLT2 inhibitor, gliflozin, heart failure, diabetes, HFpEF

## Abstract

Heart failure with preserved ejection fraction (HFpEF) is a complex clinical syndrome with high morbidity and increasing socio-economic burden, compounded by the lack of effective treatment options available to treat this disease. Sodium-glucose cotransporter-2 (SGLT2) inhibitors have previously been shown to improve cardiovascular and renal outcomes in patients with type 2 diabetes and patients with heart failure with reduced ejection fraction (HFrEF). Recent major clinical trials with SGLT2 inhibitors, both empagliflozin and dapagliflozin, have now demonstrated improved cardiovascular outcomes in patients with HFpEF and a significant reduction in heart failure hospitalization. Current evidence shows a potential for cardiovascular benefits with SGLT2 inhibition that is consistent across the spectrum of ejection fraction, age, New York Heart Association (NYHA) functional class, natriuretic peptide levels and diabetes status. Although the cardioprotective mechanisms behind SGLT2 inhibition remain unclear, ongoing clinical studies aim to clarify the role of SGLT2 inhibitors on biomarkers of cardiac metabolism, diastolic function and exercise capacity in HFpEF. This article analyzes current clinical evidence from randomized controlled trials and meta-analyses and explores the potential cardioprotective mechanisms of SGLT2 inhibitors, while also looking towards the future of SGLT2 inhibition in HFpEF.

## 1. Introduction

Heart failure with preserved ejection fraction (HFpEF) is a complex clinical syndrome characterized by left ventricular ejection fraction (LVEF) ≥50% and elevated left ventricular filling pressures at rest or during exercise [1]. HFpEF is the most common type of heart failure (HF) in patients over 65 years [2] and accounts for approximately 40–50% of incident HF in the community [3]. Indeed, the incidence of HFpEF has been shown to be rising [4]. This has been associated with increased morbidity, higher rates of hospitalization and rising healthcare costs, leading to an increased burden of disease for patients, caregivers and healthcare institutions [5, 6]. As such, HFpEF is a critical public health problem associated with an increasing economic burden, compounded by the presence of higher comorbidity and by the scarcity of prognosis-modifying therapies for HFpEF in comparison to heart failure with reduced ejection fraction (HFrEF) [5, 7, 8].

Treatment of HFpEF is traditionally based on lifestyle interventions and the management of comorbidities such as diabetes, obesity, hypertension and atrial fibrillation [1]. The medication classes that improve outcomes at lower LVEF ranges have not been proven as efficacious at preserved LVEF ranges, decreasing HF hospitalizations in HFpEF, but not all-cause or cardiovascular mortality [1, 9]. Of these classes, the glycosuric sodium-glucose cotransporter-2 (SGLT2) inhibitors have been shown to have beneficial cardiovascular and renal effects in several clinical trials, independent of diabetes status [10, 11, 12, 13, 14, 15, 16, 17], with proven reductions in HF hospitalizations and cardiovascular death in patients with HFrEF [11, 12]. Once the potential of SGLT2 inhibitors in ameliorating cardiovascular outcomes in HFrEF became apparent, several clinical trials were designed to understand the effect of this drug class in HFpEF [18, 19]. The results of these major clinical trials have led recent guidelines to recommend the inclusion of SGLT2 inhibitors as optimal medical therapy for HFrEF [20].

This review aims to describe the current clinical evidence of SGLT2 inhibition in HFpEF (with a focus on recent randomized controlled trials and meta-analyses) and briefly summarize the potential cardioprotective mechanisms of SGLT2 inhibitors while outlining ongoing research in this area.

## 2. Clinical Evidence with the Use of Gliflozins: A Journey from Diabetes to HFpEF

The cardiovascular benefits of SGLT2 inhibitors were an unexpected finding from the EMPA-REG OUTCOME (Empagliflozin Cardiovascular Outcome Event Trial in Type 2 Diabetes Mellitus Patients) trial, which showed that empagliflozin was associated with a significant reduction in the risk of HF hospitalization and cardiovascular death in patients with type 2 diabetes and established cardiovascular disease [21]. Since then multiple large-scale clinical trials showed similar results in patients with HFrEF, which then paved the way for subsequent trials in HFpEF [18, 19]. Current evidence suggests the benefit of SGLT-2 inhibition across the cardiorenal continuum, independent of diabetes status [17].

In the next chapters we will briefly review the clinical evidence for the use of gliflozins in HFpEF. The study characteristics and main results of each of the major randomized clinical trials in HFpEF are summarized in Table 1.

**Table 1.** **Summary of Major Randomized Clinical Trials of SGLT2 Inhibitors in HFpEF**.

Drug name	Trial name	Study population	Primary outcome	Main results
Sotagliflozin	SOLOIST-WHF	1222 patients (20% with LVEF >50%)	Composite of total number of CV deaths and HF exacerbations (HHF or urgent visit)	HR for composite outcome: 0.67 (95% CI 0.52–0.85)
		Age ≥18 years		HR for CV death: 0.84 (95% CI 0.58–1.22)
		Recent HHF		HR for WHF: 0.64 (95% CI 0.49–0.83)
		T2DM		
Empagliflozin	EMPEROR-Preserved	5988 patients	Composite of CV death or HHF	HR for composite outcome: 0.79 (95% CI 0.69–0.90)
		Age ≥18 years		HR for CV death: 0.91 (95% CI 0.76–1.09)
		NYHA II–IV		HR for HHF: 0.71 (95% CI 0.60–0.83)
		LVEF >40%		
	EMPERIAL-Preserved	315 patients	6MWD change after 12 weeks	Change in 6MWD: 4.0m (95% CI −5.0–13.0)
		Age ≥18 years		
		LVEF >40%		
Dapagliflozin	DELIVER	6263 patients	Composite of CV death or HF exacerbations (HHF or urgent visit)	HR for composite outcome: 0.82 (95% CI 0.73–0.92)
		Age ≥40 years		HR for CV death: 0.88 (95% CI 0.74–1.05)
		NYHA II–IV		HR for WHF: 0.79 (95% CI 0.69–0.91)
		LVEF >40% (including prior LVEF ≤40%)		
	PRESERVED-HF	324 patients	Change in KCCQ Clinical Summary Score after 12 weeks	Change in KCCQ: 5.8 points (95% CI 2.3–9.2)
		Age ≥18 years		
		NYHA II–IV		
		LVEF ≥45%		
Canagliflozin	CANONICAL	82 patients	Change in body weight and plasma BNP levels after 24 weeks	Reduction in body weight with canagliflozin (*p* = 0.019)
		Age ≥65 years		No significant change in BNP levels
		LVEF ≥50%		
		T2DM		
	CHIEF-HF	476 patients (276 with HFpEF)	Change in KCCQ Total Symptom Score after 24 weeks	Change in KCCQ: 4.3 points (95% CI 0.8–7.8)
		Age ≥18 years		Change in KCCQ (HFpEF group): 4.5 points (95% CI −0.3–9.4)
		History of HF (LVEF ≥40% for HFpEF group)		
Ertugliflozin	VERTIS-CV	8246 patients (1007 patients with LVEF >45%)	Composite of CV death, non-fatal MI or non-fatal stroke	HR for composite outcome: 0.97 (95% CI 0.85–1.11)
		Age ≥40 years		HR for first HHF: (LVEF >45%): 0.86 (95% CI 0.58–1.29)
		T2DM		
Luseogliflozin	MUSCAT-HF (luseogliflozin vs. voglibose)	190 patients	Change in plasma BNP levels after 12 weeks	Change in ratio of BNP levels: 0.93 (95% CI, 0.78–1.10)
		Age ≥20 years		
		LVEF >45%		
		T2DM		
Ipragliflozin	EXCEED	68 patients	Change in E/e’ and e’ after 24 weeks	Change in E/e’: –0.04 (95% CI –1.3–1.2)
		Age ≥20 years		Change in e’: 0.3 cm/s (95% CI –0.9–0.3)
		LVEF ≥50%		
		T2DM		

BNP, B type natriuretic peptide; CI, confidence interval; CV, cardiovascular; HF, heart failure; HHF, hospitalization for heart failure; HFpEF, heart failure with preserved ejection fraction; HR, hazard ratio; KCCQ, Kansas City Cardiomyopathy Questionnaire; LVEF, left ventricular ejection fraction; MI, myocardial infarction; NYHA, New York Heart Association; SGLT2, sodium-glucose cotransporter-2; T2DM, type 2 diabetes; WHF, worsening heart failure; 6MWD, six minute walking distance.

### 2.1 HF Hospitalizations and Acute HF Exacerbations

The EMPEROR-Preserved trial was the first clinical trial to show a clear benefit in a composite outcome of cardiovascular mortality and HF hospitalization in patients with HFpEF. This trial analyzed the effect of empagliflozin versus placebo in a group of 5988 patients with symptomatic HF and LVEF >40%. There was a 21% relative risk reduction for the composite primary outcome (hazard ratio [HR] 0.79; 95% confidence interval [CI] 0.69–0.90), largely due to a 29% lower relative risk of HF hospitalization (HR 0.71; 95% CI 0.60–0.83). This effect was found to be strongest for the subgroup of patients with LVEF <50% (i.e., the mildly reduced HF [HFmrEF] subgroup), who comprised approximately one-third of the trial population (HR 0.71; 95% CI 0.57–0.88) [18]. Empagliflozin also showed a clinical benefit independent of baseline N-terminal pro-B type natriuretic peptide (NT-proBNP) or high-sensitivity cardiac troponin T levels [22]. A pooled meta-analysis of data from both EMPEROR trials found that the benefit of empagliflozin was consistent in patients with reduced and preserved LVEF, although the authors found a potential attenuation of effect in a sub-group of patients with LVEF ≥65% [23]. When considering potential interactions with other HF medications, the effect of empagliflozin in reducing first and total HF hospitalizations persisted regardless of concomitant treatment with mineralocorticoid antagonists [24].

More recently, the DELIVER trial, a phase III randomized clinical trial studying the effect of dapagliflozin on patients with preserved or mildly reduced LVEF (LVEF >40%), showed similar results [19]. DELIVER was a multicenter, event-driven, double-blind, randomized controlled trial that randomized 6263 patients to treatment with dapagliflozin 10 mg once daily or placebo. Eligible patients were at least 40 years of age, had stabilized HF with LVEF >40% (including patients with improved LVEF from ≤40%), and had evidence of structural heart disease and elevated natriuretic peptides. Patients could be enrolled as outpatients or during hospitalization for HF (after stabilization). The trial found a statistically significant risk reduction of 18% in the primary composite endpoint of time to cardiovascular death or worsening HF with dapagliflozin (HR 0.82; 95% CI 0.73–0.92), mostly driven by a reduction in worsening HF events with no effect on cardiovascular mortality [19]. This effect was found to be consistent in a subgroup analysis comparing patients with LVEF ≥60% and LVEF <60%, showing no attenuation of benefit in patients with higher LVEF [25]. Similarly, this benefit was also found to be maintained in the subgroup of patients with improved LVEF from ≤40% [19]. Patients with atrial fibrillation (particularly paroxysmal atrial fibrillation) were found to be at greater risk of suffering the primary endpoint (mostly due to HF hospitalizations), however, treatment with dapagliflozin (as compared to placebo) was found to be consistent regardless of the presence or absence of atrial fibrillation [26]. Benefit of dapagliflozin treatment was also found to be consistent and irrespective of frailty class [27], age [28], body mass index [29]or New York Heart Association (NYHA) functional class [30].

In the VERTIS CV (Evaluation of Ertugliflozin Efficacy and Safety Cardiovascular Outcomes Trial) trial, which studied the effect of ertugliflozin versus placebo in a group of 8246 patients with type 2 diabetes mellitus and established atherosclerotic cardiovascular disease, ertugliflozin was found to reduce the risk of first and total HF hospitalization (HR 0.70; 95% CI 0.54–0.90) [14]. The effects of ertugliflozin were similar in patients with known HFrEF and HFpEF, although it must be noted that only 23.7% of patients included in the trial had HF, 68% of whom had HFpEF (defined as LVEF >45%) [31].

Sotagliflozin is a dual SGLT-2 and SGLT-1 inhibitor, developed for the treatment of type 1 and type 2 diabetes mellitus. Due to its effects on SGLT-1 inhibition, sotagliflozin has an additional glucose-lowering mechanism by delaying the gastrointestinal absorption of glucose [32]. In the SCORED (Effect of Sotagliflozin on Cardiovascular and Renal Events in Patients with Type 2 Diabetes and Moderate Renal Impairment Who Are at Cardiovascular Risk) trial, 10,584 patients with chronic kidney disease and type 2 diabetes mellitus were randomized to treatment with sotagliflozin or placebo. In this trial, 31% of the patients randomized had a previous history of HF, with a median LVEF of 60%. Approximately 21% of the patients randomized had an LVEF of greater than 40%, while 19.9% of the patients presented an LVEF of less than 40% or had been hospitalized for HF within the previous two years. Patients randomized to sotagliflozin presented a lower risk of suffering the primary endpoint of total cardiovascular deaths, HF hospitalizations or urgent HF visits (HR 0.74; 95% CI 0.63–0.88) [33].

The effects of SGLT2 inhibitors in patients hospitalized with acute HF has been studied in populations including both HFrEF and HFpEF patients. The SOLOIST-WHF (Effect of Sotagliflozin on Cardiovascular Events in Patients with Type 2 Diabetes Post Worsening Heart Failure) trial was a randomized, double-blind trial in which 1222 patients with type 2 diabetes mellitus who were recently hospitalized for acute decompensated HF were randomized to treatment with sotagliflozin or placebo. Approximately 20% of the patients included had HFpEF (LVEF ≥50%). Although the trial was stopped early due to loss of funding, sotagliflozin led to a reduction in the rate of the primary composite endpoint of total cardiovascular deaths, HF hospitalizations and urgent HF visits (HR 0.67; 95% CI 0.52–0.85) over a median of nine months follow-up [13]. The EMPULSE trial included 530 patients hospitalized for acute HF, who were randomized as soon as possible after stabilization (before hospital discharge) to treatment with empagliflozin or placebo and followed for up to 90 days. Approximately 32% of the patients included presented an LVEF >40%. The primary outcome was clinical benefit assessed using a win-ratio analysis, which included a composite of death from any cause, number of HF events, time to first HF event, or a change in the Kansas City Cardiomyopathy Questionnaire (KCCQ) Total Symptom Score of ≥5 points. In this trial, more patients treated with empagliflozin had clinical benefit compared with placebo (stratified win ratio 1.36; 95% CI 1.09–1.6), an effect which was observed regardless of ejection fraction. Finally, the DELIVER trial showed that in the 654 patients included who were recently hospitalized (post-stabilization or within 30 days after discharge), dapagliflozin was also found to be effective with a 22% reduction in the primary outcome (HR 0.78; 95% CI 0.60–1.03), without an increase in adverse events [34].

In a pooled analysis of the SOLOIST-WHF and SCORED trials including 11,784 patients, sotagliflozin showed a benefit in cardiovascular outcomes across the spectrum of ejection fractions, including HFpEF [35, 36]. However, these initial data were limited as patients with HFpEF comprised a very small subgroup of both the SOLOIST-WHF and SCORED trials, making it difficult to draw firm conclusions about the effects of SGLT-2 inhibition in HFpEF from these trials [13].

Recent meta-analyses have helped to demonstrate the benefits of SGLT2 inhibition in HFpEF, mainly due to a reduced incidence of HF hospitalization as well as in the composite outcome of cardiovascular death or HF hospitalization with the use of SGLT2 inhibitors [37, 38, 39]. These meta-analyses also showed evidence for a reduced incidence of first HF hospitalization with SGLT2 inhibitors [38] and persistent benefit in a subgroup of patients with stage 3–4 chronic kidney disease and HFpEF [40].

### 2.2 Cardiovascular and All-Cause Mortality

Contrary to the results regarding HF hospitalizations, SGLT2 inhibitors have not shown a significant decrease in cardiovascular death in any of the major randomized clinical trials studying HFpEF patients [14, 18, 19, 36].

However, along with the results from the DELIVER trial, a patient-level pooled meta-analysis was published using results from two trials (DELIVER and DAPA-HF [Dapagliflozin and Prevention of Adverse Outcomes in Heart Failure]) testing the use of dapagliflozin in patients with HF and across the range of left ventricular function (namely LVEF >40% and ≤40%). This meta-analysis pooled results from 11,007 participants who were randomized to treatment with dapagliflozin or placebo, and found that dapagliflozin reduced the risk of death from cardiovascular causes, death from any cause and total HF hospitalizations, irrespective of LVEF [25]. When considering the primary composite endpoint of the DAPA-HF and DELIVER trials (that is, time to HF hospitalization or death from cardiovascular causes), dapagliflozin was found to reduce the risk of the primary outcome by 22% [25]. These results seem to be contrary to the results of the pooled EMPEROR trials, a *post hoc* analysis which found that treatment with empagliflozin appeared to be attenuated in patients with LVEF ≥65% [23]. This subgroup, however, comprised only 10% of the total trial population, and as such these results may be imprecise for patients with higher LVEF. Notwithstanding, the recent results from the dapagliflozin trials, comprising data from a larger population base, may serve to assuage the concerns from the pooled EMPEROR trials regarding the efficacy of SGLT2 inhibitors in patients with supra-normal LVEF (≥65%).

In a prespecified meta-analysis including results from the 12,251 patients included in the EMPEROR-Preserved and DELIVER trials, SGLT2 inhibitors reduced the primary composite outcome of cardiovascular death or first hospitalization for HF, without evidence of significant heterogeneity between trials. Both components of the primary outcome had consistent reductions with SGLT2 inhibitor use, with demonstrated reductions in cardiovascular death, first HF hospitalization and worsening HF events when each outcome was considered separately. No significant difference in all-cause death was found [41].

When including results from five outcome trials with SGLT2 inhibitors across the range of LVEF (DAPA-HF, EMPEROR-Reduced, DELIVER, EMPEROR-Preserved and SOLOIST), the use of SGLT2 inhibitors as compared with placebo showed a reduction in the risk of cardiovascular death or HF hospitalization over an average of 23 months of follow-up, independent of LVEF, with a number needed to treat of 25 [41].

### 2.3 Quality of Life and Exercise Capacity in HFpEF Patients

Several trials aimed to provide insights into the effect of SGLT2 inhibitors on overall health status and exercise capacity in patients with HFpEF. Recent data from the EMPERIAL (Effect of EMPagliflozin on ExeRcise ability and HF symptoms In patients with chronic heArt faiLure) trials analyzed the effect of empagliflozin after twelve weeks of treatment on health status in both HFrEF and HFpEF patients, with and without type 2 diabetes, but no significant differences in health status outcomes were observed in either trial [42]. However, in the DELIVER trial, dapagliflozin was associated with a statistically significant improvement in the KCCQ Total Symptom Score, with a mean improvement of 2.4 points at eight months (95% CI 1.5–3.4), although the magnitude of this improvement was mild [19].

The effect of dapagliflozin on HF-related health status was also evaluated in a previous trial, PRESERVED-HF, in which 324 patients with symptomatic HF (NYHA II-IV), elevated natriuretic peptides and LVEF ≥45% were randomized to 12 weeks of treatment with dapagliflozin or placebo [43]. Change in the KCCQ Clinical Summary Score after treatment was evaluated as the primary endpoint. The authors found that treatment with dapagliflozin led to a 5.8 point improvement in the KCCQ Clinical Summary Score (95% CI 2.3–9.2), mainly due to improvements in the KCCQ total symptom scores and physical limitations scores. Dapagliflozin was also associated with an increase in six-minute walking distance (6MWD). These results may thereby show evidence for an early benefit in health status and physical function with SGLT2 inhibition [43].

Amidst the COVID19 pandemic, the CHIEF-HF investigators designed a novel type of clinical trial, conducted remotely, which studied the effect of canagliflozin on health status outcomes in patients with HF, regardless of LVEF or presence of type 2 diabetes [44]. Although the trial was stopped early due to shifting sponsor priorities, the primary outcome of change in the KCCQ total symptom score was met after 12 weeks of treatment, with a 4.3 point increase in the KCCQ score in the intervention arm (95% CI 0.8–7.8). A 4.5 point increase in the KCCQ score was shown in the HFpEF subgroup, although this value was not statistically significant (95% CI –0.3–9.4) [44].

A recent meta-analysis considering differences in exercise capacity with SGLT2 inhibitors in HFpEF found that treatment with this drug class did not lead to a difference in six-minute walking distance [39]. Although the currently available results only show a mild benefit with SGLT2 inhibitor use when considering quality of life outcomes, several major international randomized clinical trials are further studying the effect of treatment with dapagliflozin (NCT03877224 and NCT04730947) and empagliflozin (NCT03753087) on the exercise capacity of HFpEF patients.

### 2.4 Echocardiographic Parameters and Cardiovascular Biomarkers

The effects of SGLT2 inhibitors on diastolic function has been established as one of the potential mechanisms for cardiovascular benefit in this drug class, particularly in experimental models [45, 46, 47]. In the EMPA-REG-OUTCOME trial, treatment with empagliflozin was associated with decreased left ventricular mass index and improved diastolic function as measured by e’ [48]. In patients with stable HF, including HFpEF, dapagliflozin has been shown to decrease E/e’ ratios as well as improved global longitudinal strain [49, 50]. When considering HFpEF patients in particular, a study analyzing the effect of the SGLT2 inhibitors luseogliflozin, empagliflozin and tofogliflozin on left ventricular function in patients with type 2 diabetes and HFpEF showed that these drugs led to a significant decrease in E/A ratios and E/e’ ratios after treatment [51]. However, in another study including patients with type 2 diabetes and HFpEF (LVEF ≥50%), ipragliflozin was not found to have a significant effect on diastolic function when compared with conventional treatment [52]. Ongoing studies (NCT04739215 and NCT04475042) may help further clarify the effect of SGLT2 inhibition on diastolic function in HFpEF patients.

Empagliflozin and dapagliflozin both showed a consistent benefit in HF hospitalizations regardless of baseline natriuretic peptide levels [22, 53], with dapagliflozin also showing a greater absolute effect in patients with higher baseline NT-proBNP levels [53]. When regarding specific effects of SGLT2 inhibitors on cardiovascular biomarkers, in EMPEROR-Preserved, empagliflozin led to a modest reduction in NT-ProBNP levels by approximately 7% over 100 weeks of treatment. However, most trials did not find a significant reduction in natriuretic peptide levels with SGLT2 inhibitors [54, 55, 56, 57].

In a meta-analysis considering differences in echocardiographic parameters, biomarkers and adverse events between the SGLT2 inhibitor and placebo groups, SGLT2 inhibitors significantly reduced the E/e’ ratio and the incidence of adverse events in patients with HFpEF, but did not affect natriuretic peptide levels [39].

### 2.5 Real-World Eligibility for Dapagliflozin and Empagliflozin in HFpEF

Real-world evidence for the cardiovascular outcomes of dapagliflozin and empagliflozin in HFpEF may still be scarce, but it is important to consider the generalizability of the results of these randomized clinical trials in a real-world population. The main eligibility criteria for the DELIVER, EMPEROR-Preserved and SOLOIST trials are summarized in Table 2.

**Table 2.** **Eligibility Criteria for SGLT2 Inhibitor Trials in HFpEF**.

Trial name		SOLOIST-WHF	EMPEROR-Preserved	DELIVER
Age		18–85 years	≥18 years	≥40 years
LVEF		-	>40%	>40%
Prior LVEF ≤40%		-	No	Yes
T2DM diagnosis		Required	Not required	Not required
Current HF hospitalization		Included	Not included	Included
NYHA functional class		-	II–IV	II–IV
HF duration		≥3 months	≥3 months	≥6 weeks
Echocardiographic evidence of structural heart disease		Not required	LA enlargement or LV hypertrophy (not required for inclusion)	LA enlargement or LV hypertrophy required
Natriuretic peptides	AF absent	BNP ≥150 pg/mL or NT-proBNP ≥600 pg/mL	NT-proBNP ≥300 pg/mL	NT-proBNP ≥300 pg/mL
	AF present	BNP ≥450 pg/mL or NT-proBNP ≥1800 pg/mL	NT-proBNP ≥900 pg/mL	NT-proBNP ≥600 pg/mL
${\mathrm{eGFR}}^{1}$		≥30 mL/min/1.73 $m^{2}$	≥20 mL/min/1.73 $m^{2}$	≥25 mL/min/1.73 $m^{2}$
Recent ACS		Excluded (3 months)	Excluded (90 days)	Excluded (12 weeks)
Recent coronary revascularization		Excluded (1 month)	Excluded (90 days)	Excluded (12 weeks)

^1^ Calculated using the MDRD formula for SOLOIST-WHF and the CKD-EPI formula for EMPEROR-Preserved and DELIVER. AF, atrial fibrillation; BNP, B type natriuretic peptide; eGFR, estimated glomerular filtration rate; HF, heart failure; HFpEF, heart failure with preserved ejection fraction; LA, left atrium; LV, left ventricle; LVEF, left ventricular ejection fraction; NT-proBNP, N-terminal pro-B type natriuretic peptide; NYHA, New York Heart Association; SGLT2, sodium-glucose cotransporter-2; T2DM, type 2 diabetes.

One study used data from the Swedish HF registry (SwedeHF) to assess the eligibility of a real-world population for treatment with dapagliflozin or empagliflozin according to the selection criteria of the DELIVER or EMPEROR-Preserved trials respectively [58]. When applying strict trial criteria, 30% of HFpEF patients were eligible for treatment according to the DELIVER criteria and 32% were eligible according to the EMPEROR-Preserved criteria, mainly limited by HF duration and NT-proBNP levels. However, when considering the differences between eligible and non-eligible patients, the authors found that eligible patients were more likely to be older and to have more severe HF with higher NYHA functional class, higher NT-proBNP levels and longer HF duration than non-eligible patients [58]. Real-world evidence may not be as striking as the results from randomized clinical trials, perhaps because SGLT2 inhibitors may be more likely to benefit sicker patients.

Nevertheless, it must be remembered that patients with HFpEF often present several comorbidities, aside from type 2 diabetes, which may broaden the population eligible for SGLT2 inhibition according to strict trial criteria. One such comorbidity which has been gaining emphasis in recent trials is chronic kidney disease, due to favorable renal outcomes in several landmark trials [59, 60, 61]. The CREDENCE (Canagliflozin and Renal Events in Diabetes with Established Nephropathy Clinical Evaluation) trial included patients with type 2 diabetes, chronic kidney disease (with an estimated glomerular filtration rate [eGFR] between 30 and 90 mL/min/1.73 $m^{2}$) and albuminuria, and showed a decreased risk of kidney failure and progression of kidney disease in the canagliflozin group [10]. More recently, the DAPA-CKD (Dapagliflozin and Prevention of Adverse Outcomes in Chronic Kidney Disease) trial included 4094 patients, irrespective of diabetes status, who presented an eGFR between 25 and 75 mL/min/1.73 $m^{2}$ (CKD-EPI formula) and a urinary albumin-to-creatinine ratio ≥200 mg/g. The trial was stopped early due to efficacy, with a significantly lower rate of the composite outcome of a sustained decline in the eGFR of at least 50%, end-stage kidney disease or death from renal causes in the dapagliflozin group [15]. Finally, the results of the EMPA-KIDNEY trial (NCT03594110), stopped early due to positive interim efficacy, have been highly anticipated. This trial included two groups of patients with evidence of chronic kidney disease, with or without proteinuria: one group including patients with an eGFR between 20 and 45 mL/min/1.73 $m^{2}$ (CKD-EPI formula); and the other including patients with an eGFR between 45 and 90 mL/min/1.73 $m^{2}$ as well as a urinary albumin-to-creatinine ratio ≥200 mg/g (or protein:creatinine ratio ≥300 mg/g) [62]. In this trial, empagliflozin reduced the risk of the primary outcome (a composite of kidney disease progression or death from cardiovascular causes) by 28% (HR 0.72, 95% CI 0.78–0.95), with consistent results between subgroups and greater risk reduction in patients with higher urinary albumin-to-creatinine ratios [63]. However, there were no significant differences between the empagliflozin and placebo groups with respect to HF hospitalizations or death from cardiovascular causes, likely due to a low number of cardiovascular events during the trial [63].

In this manner, patients with HFpEF may be eligible for cardioprotective treatment with SGLT2 inhibitors when considering the presence of nephropathy or their diabetes status, and not only according to HFpEF criteria.

## 3. Potential Cardioprotective Mechanisms of SGLT2 inhibition in HFpEF 

As has been discussed in previous chapters, SGLT2 inhibitors are the first drug class that has been shown to clearly improve cardiovascular outcomes in patients with HFpEF [18, 19]. However, the cardioprotective mechanisms behind SGLT2 inhibition remain unclear, and several potential mechanisms have been proposed for the beneficial cardiovascular and renal effects of these drugs, as will be discussed in this section. Furthermore, multiple studies are currently underway to further elucidate the potential benefits and mechanisms of this drug class in HF patients, the main characteristics of which may be found in Table 3.

**Table 3.** **Ongoing or ${\mathrm{Unpublished}}^{1}$ Trials of SGLT2 Inhibitors in HFpEF**.

Trial number (name)	Therapy	Population	Primary outcome	Expected enrolment	Current ${\mathrm{status}}^{1}$
NCT04071626 (EMMED-HF)	Ertugliflozin	LVEF >50%	Change in peak oxygen uptake as measured by peak VO2 (mL/kg/min)	52	Recruiting
		BMI 29–42 kg/$m^{2}$			
		T2D or insulin resistance			
NCT03877224 (DETERMINE-Preserved)	Dapagliflozin	LVEF >40%	Change from baseline KCCQ-TSS and KCCQ-PLS scores	504	Completed
		Evidence of structural heart disease	Change from baseline 6MWD		
NCT04730947	Dapagliflozin	LVEF ≥50%	Change in PCWP during exercise	46	Recruiting
		BMI ≥30 kg/$m^{2}$			
		Elevated PCWP during exercise (≥25 mmHg)			
NCT03753087	Empagliflozin	LVEF ≥50% + T2DM	Change from baseline 6MWD	70	Completed
NCT04739215 (CARDIA-STIFF)	Dapagliflozin	LVEF ≥50% + T2DM	Change from baseline LV stiffness constant (S+) during exercise	62	Recruiting
		Recent HF hospitalization (6 months)	Change from baseline PICP levels		
		Indication for cardiac catheterization			
NCT04475042 (STADIA-HFpEF)	Dapagliflozin	LVEF ≥50% + LVEDV <97 mL/$m^{2}$	LV e’	26	Recruiting
		Evidence of LV diastolic dysfunction	E/e’ LV end-diastolic volume index		
		Cardiac MRI extracellular matrix volume <29%			
NCT05138575 (SAK HFpEF)	Empagliflozin ± potassium nitrate	LVEF ≥50%	Submaximal exercise endurance	53	Recruiting
		Evidence of elevated diastolic filling pressures			
NCT03332212 (EMPA-VISION)	Empagliflozin	LVEF ≤40% (Cohort A)	Change from baseline PCr/ATP ratio at rest	72	Completed
		LVEF ≥50% (Cohort B)			

^1^ At the time of article submission. BMI, body mass index; HF, heart failure; HFpEF, heart failure with preserved ejection fraction; KCCQ-TSS, Kansas-City Cardiomyopathy Questionnaire-Total Symptom Score; KCCQ-PLS, Kansas-City Cardiomyopathy Questionnaire-Physical Limitation Score; LV, left ventricle; LVEDV, left ventricle end-diastolic volume; LVEF, left ventricular ejection fraction; MRI, magnetic resonance imaging; PICP, pro-collagen type I C-terminal propeptide; PCWP, pulmonary capillary wedge pressure; PCr/ATP, phosphocreatine/adenosine triphosphate; SGLT2, sodium-glucose cotransporter-2; T2DM, type 2 diabetes; VO2, oxygen consumption; 6MWD, 6-minute walking distance.

### 3.1 Renal Mechanisms in SGLT2 Inhibition

SGLT2 inhibitors block SGLT2 cotransporters in the proximal tubules of the kidney, thereby inhibiting renal glucose reabsorption and causing glycosuria, leading to a reduction in blood glucose levels and a reduction in HbA1c of about 0.5–1.0% in patients with diabetes, while these effects are attenuated in non-diabetic patients [64]. Aside from glycosuria, SGLT2 inhibitors were also thought to increase the excretion of urinary sodium by decreasing the reabsorption of approximately 40% of urinary sodium in the proximal tubule as well as by a mild osmotic effect [65]. However, this diuretic effect is not sustained, mainly due to the activation of adaptive renal mechanisms to reduce free water clearance, and as such may not lead to a significant change in urinary sodium concentrations [66].

A recent study which evaluated the diuretic effects of empagliflozin found that SGLT2 inhibition had a modest natriuretic effect with a synergistic natriuretic effect when combined with loop diuretics [67]. This natriuretic effect, contrary to traditional diuretics, occurs without the activation of the neurohormonal or renin–angiotensin–aldosterone systems and without increased excretion of potassium or magnesium [67]. Unlike loop diuretics, SGLT2 inhibitors do not inhibit intravascular volume sensing by the macula densa, and so do not lead to a compensatory increase in renin secretion or intraglomerular pressures [65]. This signifies that SGLT2 inhibitors do not lead to the braking phenomenon often seen with loop diuretics, where the chronic use of loop diuretics leads to the increased reabsorption of sodium by the distal nephron with a secondary decrease in natriuresis [68]. Furthermore, SGLT2 inhibitors also interact with sodium-hydrogen exchangers in the kidneys by inhibiting their action [69]. This is significant as sodium-hydrogen exchanger activity is increased in patients with HF and may be responsible in part for increased diuretic resistance in HF [70]. As such, SGLT2 inhibitors may offer a significant advantage to loop diuretics in the management of volume status in HF patients, as also suggested in studies in acute HF [71].

Additionally, increased renal sympathetic activity appears to be an important mechanism in the progression of HF due to increased activation of the renin-angiotensin system [72, 73]. Common comorbidities in HF such as diabetes and obesity are associated with chronic activation of the sympathetic nervous system [74]. In an experimental model, SGLT2 inhibition with dapagliflozin was shown to lead to decreased renal sympathetic activity in hypertensive mice, with lowered blood pressure, reduced weight gain, lower levels of inflammatory cytokines and improved endothelial function [75]. Therefore, SGLT2 inhibitors may counteract renal sympathetic overactivity in a manner which is functionally similar to renal denervation [76].

### 3.2 Role of SGLT2 Inhibition with HFpEF-Associated Comorbidities

SGLT2 inhibitors may help to treat many of the comorbidities associated with HFpEF through increased natriuresis, glycosuria, and osmotic diuresis, leading to consequent reductions in body weight, blood pressure, blood glucose levels, uric acid levels and lipid profiles [69, 77, 78, 79].

A recent meta-analysis showed that SGLT2 inhibitors led to a significant reduction in body weight and body mass index in non-diabetic overweight or obese patients [80]. This weight loss appears to be induced by the glycosuric effects of SGLT2 inhibitors [81], and the resulting decrease in adiposity may lead to a reduction in the low-grade inflammation associated with fat deposition. For instance, in mice, empagliflozin has been shown to promote the utilization of fat by increasing the browning of adipose tissue. The increase in brown fat was associated with an increase in energy expenditure and was also found to induce the alternate activation of anti-inflammatory macrophages in adipose tissues [82, 83]. Additionally, SGLT2 inhibitors have been found to reduce epicardial adipose tissue [84, 85], which is an independent marker of cardiovascular risk, particularly in patients with HFpEF [86, 87]. Results from the EMPA-TROPISM study suggest that the reduction in epicardial adipose fat seen with empagliflozin may lead to a reduction in proinflammatory adipokines, which may in turn be associated with decreased aortic stiffness and decreased interstitial myocardial fibrosis in nondiabetic HFrEF patients [87, 88].

The cardiovascular benefits of SGLT2 inhibitors are preserved across the spectrum of renal function, even though the efficacy of glucose reduction is diminished at lower glomerular filtration rates [17, 89, 90]. In this manner, the favorable effects of SGLT2 inhibitors in HFpEF are not fully explained by the control of the metabolic comorbidities associated with the HFpEF syndrome and appear to be consistent across cholesterol levels [91] and independent of blood pressure reduction [90, 92, 93, 94].

Anemia is a common comorbidity in HFpEF and is frequently associated with poorer outcomes [95, 96]. In a mediation analysis of the EMPA-REG OUTCOME trial, investigators found that changes in hemoglobin and hematocrit levels mediated the effect of empagliflozin on cardiovascular mortality [97]. Smaller mediation effects were also noted with reduced uric acid levels and improved glucose metabolism in the empagliflozin group [97]. In a substudy of the EMPA-HEART (Effects of Empagliflozin on Cardiac Structure in Patients With Type 2 Diabetes) CardioLink-6 randomized clinical trial, empagliflozin treatment over six months led to an increase in plasma erythropoietin levels, increased hematocrit and reduced ferritin levels in patients with type 2 diabetes and coronary disease [98].

### 3.3 Cardiovascular Effects of SGLT2 Inhibition

The use of SGLT2 inhibitors has a clear impact on cardiovascular outcomes, in particular when considering their impact on HF hospitalizations. Recent evidence supports a pleiotropic and multifaceted effect of SGLT2 inhibition, with several studies showcasing positive effects on diastolic function and cellular metabolism as further detailed below [99].

#### 3.3.1 Inflammation and Endothelial Dysfunction

HFpEF is increasingly thought to develop in the context of a proinflammatory state driven by the presence of comorbidities such as obesity, diabetes mellitus, sleep apnea and hypertension [100, 101], which in turn causes cardiac microvascular endothelial inflammation [100]. This microvascular inflammation then leads to dysfunction of adjacent cardiomyocytes, which leads to increased cardiomyocyte stiffness and interstitial fibrosis, causing consequent left ventricular diastolic dysfunction [100, 102]. Endothelial dysfunction appears to be characterized not only by increased inflammation, but also by decreased nitric oxide production and increased oxidative stress [102, 103]. This paradigm shift in HFpEF pathophysiology set the stage for research into drugs that may counteract endothelial dysfunction in HFpEF, where is growing evidence that SGLT2 inhibitors may help to ameliorate endothelial dysfunction [45, 104, 105, 106].

Dapagliflozin has also been associated with diastolic function improvement in rats, potentially due to a reduction in the expression of markers of endothelial activation, cardiac inflammation and cardiac fibrosis [45]. Meanwhile, empagliflozin was shown to increase nitric oxide production and reduce oxidative stress in a cellular model of endothelial dysfunction, leading to the preservation of cardiomyocyte relaxation and contraction [105]. Furthermore, in experimental models, SGLT2 inhibitors may also lead to reduced hypertrophy and fibrosis by reducing adipocyte hypertrophy and inflammation and improving epicardial adipose tissue dysfunction [107].

Additionally, SGLT2 inhibitors simultaneously reduce uric acid levels and the production of advanced glycation end products, both of which are associated with oxidative stress and inflammation at the endothelial level [99, 108]. SGLT2 inhibitors are also capable of lowering circulating inflammatory markers [108, 109], although they have not been shown to lead to a significant change in natriuretic peptide levels in HF patients [55].

On the molecular level, SGLT2 inhibitors have been shown to have a direct anti-inflammatory effect on the heart through attenuation of the nucleotide-binding domain-like receptor protein 3 (NLRP3) inflammasome in both diabetic and non-diabetic models, which leads to reduced expression of proinflammatory cytokines [110, 111]. Interestingly, empagliflozin appears to lead to suppression of the NLRP3 inflammasome by reducing intracellular calcium [110], which further supports the role of SGLT2 inhibition in sodium-calcium homeostasis (as will be further detailed below). Furthermore, empagliflozin has also been shown to reduce pro-inflammatory cytokines and microvascular inflammation in murine models as well as in myocardial tissue samples from HFpEF patients [106, 112]. In one study, the authors found increased oxidative stress-dependent activation of endothelial nitric oxide synthetase (eNOS) in HFpEF myocardium, which led to increased oxidation and polymerization of protein kinase G1 alpha (PKG1α) in a pathway that could potentially contribute to cardiomyocyte stiffness. This pathway was significantly attenuated with empagliflozin [112]. Similarly, in a porcine model of HF, empagliflozin also improved nitric oxide signaling in the eNOS pathway, leading to increased titin phosphorylation and decreased cardiomyocyte stiffness [46]. The effects of empagliflozin on cardiac myofilament phosphorylation have also been described in a murine model [47].

The cellular effects of SGLT2 inhibitors have been further supported in a machine learning model, where empagliflozin was found to modulate cardiomyocyte oxidative stress, cardiomyocyte stiffness, extracellular matrix remodeling, cardiac hypertrophy as well as systemic inflammation. This artificial intelligence model also found that the effect of empagliflozin appeared to be predominantly mediated by inhibition of the sodium-hydrogen exchanger, with a smaller effect on the SGLT2 protein [113].

#### 3.3.2 Calcium and Sodium Homeostasis in the Cardiomyocyte

Due to the lack of SGLT-2 expression in cardiomyocytes, the benefits of SGLT2 inhibition on the heart cannot be ascribed to a direct effect on SGLT2 [104, 114]. As such, several direct cardiac mechanisms have been proposed, including inhibition of cardiac sodium-hydrogen exchanger 1 (NHE1) [115], inhibition of calcium/calmodulin-dependent kinase II (CaMKII) [116] and inhibition of the cardiac late sodium channel current (late $I_{\text{Na}}$) [117, 118].

Voltage-gated sodium channels play an important role in initiating the action potential in cardiomyocytes. When these sodium channels are in the inactive state, the cardiomyocytes are protected from initiating new action potentials and thereby limit electrical activity which may initiate arrythmias. However, some of these sodium channels may not become inactive, which creates a persistent sodium current, or late $I_{\text{Na}}$ [118, 119]. Some studies have suggested that induction of late $I_{\text{Na}}$ may have a significant role in the development of HF and arrythmias by prolonging the action potential, increasing calcium loading in the cardiomyocyte and generating both early and delayed afterdepolarizations [120, 121, 122, 123]. In this context, upregulation of CaMKII plays a central role in the stimulation of late $I_{\text{Na}}$, as well as in the development and progression of HF [118]. Overexpression and activation of CaMKII leads to increased diastolic calcium leak from the sarcoplasmic reticulum and increased cytosolic calcium, leading to contractile dysfunction and proarrhythmic effects [124, 125]. Furthermore, NHE1 mediates sodium influx in the cardiomyocyte and its activity is increased in the failing heart, contributing to the cellular sodium overload which is characteristic of HF [118]. Increased sodium influx leads to downstream changes in calcium loading in the sarcoplasmic reticulum, with important effects on cardiomyocyte excitation-contraction coupling [126]. Experimental studies have shown that NHE1 inhibition may help prevent the development or progression of HF [118, 127].

Empagliflozin reduced late $I_{\text{Na}}$ in human ventricular myocytes as well as cardiomyocytes from a murine HF model, and as such may inhibit HF-induced dysfunction of the sodium current [117, 128]. This effect was also confirmed with the SGLT2 inhibitors dapagliflozin and canagliflozin in the same study, potentially suggesting a class effect [117]. Moreover, empagliflozin appears to bind to the same region of the sodium channel as lidocaine and ranolazine, both of which are known sodium channel inhibitors [117]. Interestingly, in a different HFpEF murine model, direct treatment with empagliflozin did not change late $I_{\text{Na}}$, but preincubation with empagliflozin over a period of four hours reversed late $I_{\text{Na}}$ enhancement [129]. This suggests that inhibition of late $I_{\text{Na}}$ may more likely be due to inhibition of CaMKII activity and a subsequent reduction of CaMKII-dependent phosphorylation of cardiac sodium channels, rather than by a direct inhibitory effect of empagliflozin on cardiac sodium channels [128, 129]. Furthermore, in the same study, the effect of empagliflozin was inhibited in cardiomyocytes with oxidation-resistant mutations in CaMKII [129].

Additionally, empagliflozin reduced CaMKII activity in murine ventricular myocytes, and also reduced CaMKII-dependent phosphorylation of cardiac ryanodine receptor type 2 (RyR2) [116], a receptor which may potentially play an important role in the pathogenesis of cardiac arrythmias due to its function in excitation-contraction coupling [130]. Reduced CaMKII activity and RyR2 phosphorylation with empagliflozin resulted in reduced sarcoplasmic reticulum calcium leak and improved contractility in failing murine and human ventricular myocytes [116].

SGLT2 inhibitors have been shown to decrease activity of NHE1 in experimental models, directly lowering cytoplasmic sodium and calcium levels in the myocardium [131, 132]. In tissue samples from human patients, NHE1 was found to be expressed more abundantly in atrial myocytes isolated from patients with HFpEF and atrial fibrillation, as well in atrial and ventricular myocytes isolated from patients with end-stage HF, which may be due to a greater impairment in atrial contractile function in patients with atrial fibrillation and globally impaired contractility in patients with end-stage HF [115]. Empagliflozin was shown to reduce NHE1 activity in human cardiomyocytes, and as such may help to improve contractile dysfunction by reducing cellular sodium and calcium load [115].

Therefore, SGLT2 inhibitors appear to have direct cardiac effects on sodium and calcium homeostasis, and may potentially ameliorate contractile function and decrease arrythmia risk in patients with HFpEF. Considering the potential effects of SGLT2 inhibition on arrythmias, two randomized clinical trials (NCT04792190 and NCT04583813) aim to evaluate whether empagliflozin or dapagliflozin may be effective to reduce atrial fibrillation burden, both in patients who undergo catheter ablation for atrial fibrillation (DAPA-AF [NCT04792190]) or patients with diabetes mellitus or obesity with an indication for rhythm control (EMPA-AF [NCT04583813]).

#### 3.3.3 Diastolic Dysfunction and Cardiac Hemodynamics

The presence of diastolic dysfunction is one of the hallmarks of HFpEF and some studies have shown the ability of SGLT2 inhibitors to reverse adverse cardiac remodeling [46, 133, 134, 135]. In a recent randomized controlled trial, treatment with dapagliflozin was shown to significantly reduce left ventricular mass in patients with type 2 diabetes and left ventricular hypertrophy, with accompanying reductions in body weight, adipose tissue, insulin resistance and high-sensitivity C-reactive protein [133]. In a nondiabetic murine model, empagliflozin has also been shown to reduce left ventricular mass and thereby lead to reduced wall-stress and improved diastolic function on conductance catheterization, and as such may have the potential to improve cardiac hemodynamics [136]. Furthermore, empagliflozin decreased diastolic tension and increased phosphorylation of cardiac myofilament proteins in both diabetic and non-diabetic murine models, with improved diastolic function as measured by a shortened isovolumetric relaxation time and increased E/A ratio [47]. In a mouse model, dapagliflozin reduced septal and lateral e’ velocities and also showed evidence for reduced myocardial fibrosis on histology, thus showing a potential benefit in diastolic function with SGLT2 inhibition [137].

SGLT2 inhibitors may also have the potential to improve cardiac hemodynamics, primarily through the reduction of preload due to their diuretic and natriuretic effects [92]. Some studies have shown a reduction in pulmonary artery pressures as measured by an implanted CardioMEMS™ pulmonary artery pressure sensor with dapagliflozin and empagliflozin [138, 139]. Another trial studied the effect of the SGLT2 inhibitor empagliflozin on central cardiac hemodynamics in patients with HFrEF, where 70 patients were randomized to treatment with empagliflozin or placebo and submitted to exercise hemodynamic testing at baseline and after 12 weeks of treatment [140]. This study found that treatment with empagliflozin led to a significant decrease in pulmonary capillary wedge pressure, but did not lead to a significant change in the primary endpoint (ratio of pulmonary capillary wedge pressure to cardiac index at peak exercise) or in the cardiac index [140].

Two ongoing studies with robust trial designs, CARDIA-STIFF (NCT04739215) and STADIA-HFpEF (Stratified Treatment to Ameliorate Diastolic Left Ventricular Stiffness in Heart Failure With Preserved Ejection Fraction; NCT04475042) [141] should help to clarify the effect of dapagliflozin on diastolic HF. The CARDIA-STIFF trial eligibility criteria include patients with a recent HFpEF decompensation and who have a clinical indication for cardiac catheterization, and as such may include a sicker patient population than is usual in HFpEF trials. Furthermore, the inclusion of collagen biomarkers may lead to an improved understanding of the underlying pathophysiology of diastolic dysfunction. STADIA-HFpEF is also distinct amongst ongoing HFpEF trials, due not only to its crossover design, but also due to including patients with “early” HFpEF without evidence of significant structural myocardial extracellular matrix remodeling [141].

#### 3.3.4 Modulation of Cardiac Energetics

Another proposed mechanism of SGLT2 inhibition on cardiomyocytes relates to their potential beneficial effects on mitochondrial function [142, 143]. Growing evidence shows that ketone bodies are favorable substrates in energy metabolism in the failing heart, due to the easier metabolism of ketone bodies compared to glycolysis and free fatty acid metabolism in hypoxic conditions [69, 144]. SGLT2 inhibitors increase the plasma levels of ketone bodies by inducing glycosuria, which decreases plasma glucose levels in the fasting state, thereby increasing glucagon levels and decreasing insulin levels, which lead to increased lipolysis in adipose tissue as well as increased carbohydrate to fat metabolism. The hyperactivation of lipolysis and decreased glucose supply lead to the increased production of ketone bodies by the liver [69, 142]. This mild, but persistent, hyperketonemia in patients undergoing treatment with SGLT2 inhibitors may lead to the preferential uptake and oxidation of β-hydroxybutyrate by cardiomyocytes, which in turn improves the efficiency of mitochondrial energy production in the failing heart in comparison with free fatty acid metabolism or glycolysis [143].

A number of trials are underway to further understand the effects of SGLT2 inhibitors on cardiac energy metabolism. The SAK HFpEF (SGLT2i and KNO3 in HFpEF) clinical trial (NCT05138575), considering the beneficial effects of empagliflozin on mitochondrial function and oxidative phosphorylation, aims to test the effects of empagliflozin on exercise capacity and skeletal muscle bioenergetics in patients with HFpEF and may further elucidate the protective mechanisms of SGLT2 inhibition on the failing heart. Similarly, the EMMED-HF (Evaluating Metabolic Mechanisms of Ertugliflozin in Diabetes & Heart Failure; NCT04071626) trial, aims to clarify the effect of ertugliflozin on cardiac metabolism as well as glucose and ketone body production after twelve weeks of treatment. Finally, the EMPA-VISION (NCT03332212) also aimed to study the effects of empagliflozin on cardiac physiology and energy metabolism in patients with HFrEF and HFpEF by measuring the change in phosphocreatine-to-adenosine triphosphate ratio using ^31^Phosphorus CMR spectroscopy [145]. Unfortunately, due to the constraints of the COVID-19 pandemic, the number of patients enrolled in the HFpEF arm was greatly reduced; therefore, this analysis is likely to be statistically underpowered.

### 3.4 Nutrient Deprivation Signaling and Autophagy

Overnutrition disease states such as type 2 diabetes and obesity are common comorbidities in HFpEF and are associated with a chronic inflammatory state [1, 3, 100, 146]. Studies have shown that autophagy, a cellular mechanism that mediates the degradation of damaged cellular components through a lysosome-dependent pathway, is impaired in overnutrition states, resulting in cellular and organ injury [147, 148, 149, 150]. Autophagy maintains cellular homeostasis through a complex mechanism dependent on multiple signaling pathways, culminating in the degradation of damaged organelles and denatured proteins through the lysosome [147, 150]. Nutrient deprivation states activate pathways that promote energy utilization and decrease energy storage, including fatty acid oxidation and resulting ketogenesis [151]. Low-energy states stimulate cellular housekeeping through autophagic flux, which reduces intracellular toxicity through the removal of lipid and glucose intermediates as well as damaged organelles [151].

SGLT2 inhibitors potentially simulate a fasting state through increased glycosuria [150]. Treatment with SGLT2 inhibitors is characterized by ketogenesis and erythrocytosis, both of which are typical responses to nutrient and oxygen deprivation [151]. It is also noteworthy that in statistical mediation analyses of large clinical trials, erythrocytosis has been identified as a consistent mediator of cardiovascular benefit with SGLT2 inhibition [97, 152]. SGLT2 inhibitors have also been shown to promote the signaling pathways associated with nutrient deprivation and hypoxia, which in turn stimulate ketogenesis, erythrocytosis and decreases in intracellular sodium [151]. The upregulation of these low-energy signaling pathways with SGLT2 inhibition also promotes autophagic flux in the heart and kidney which reduces oxidative stress, enhances mitochondrial function, suppresses proinflammatory pathways and helps to preserve cellular function and integrity [76, 150, 151]. In this way, the nutrient deprivation hypothesis may provide a unifying theory for the cardioprotective and renoprotective mechanisms behind SGLT2 inhibition [151].

### 3.5 Overview of Protective Mechanisms

**Fig. 1.**
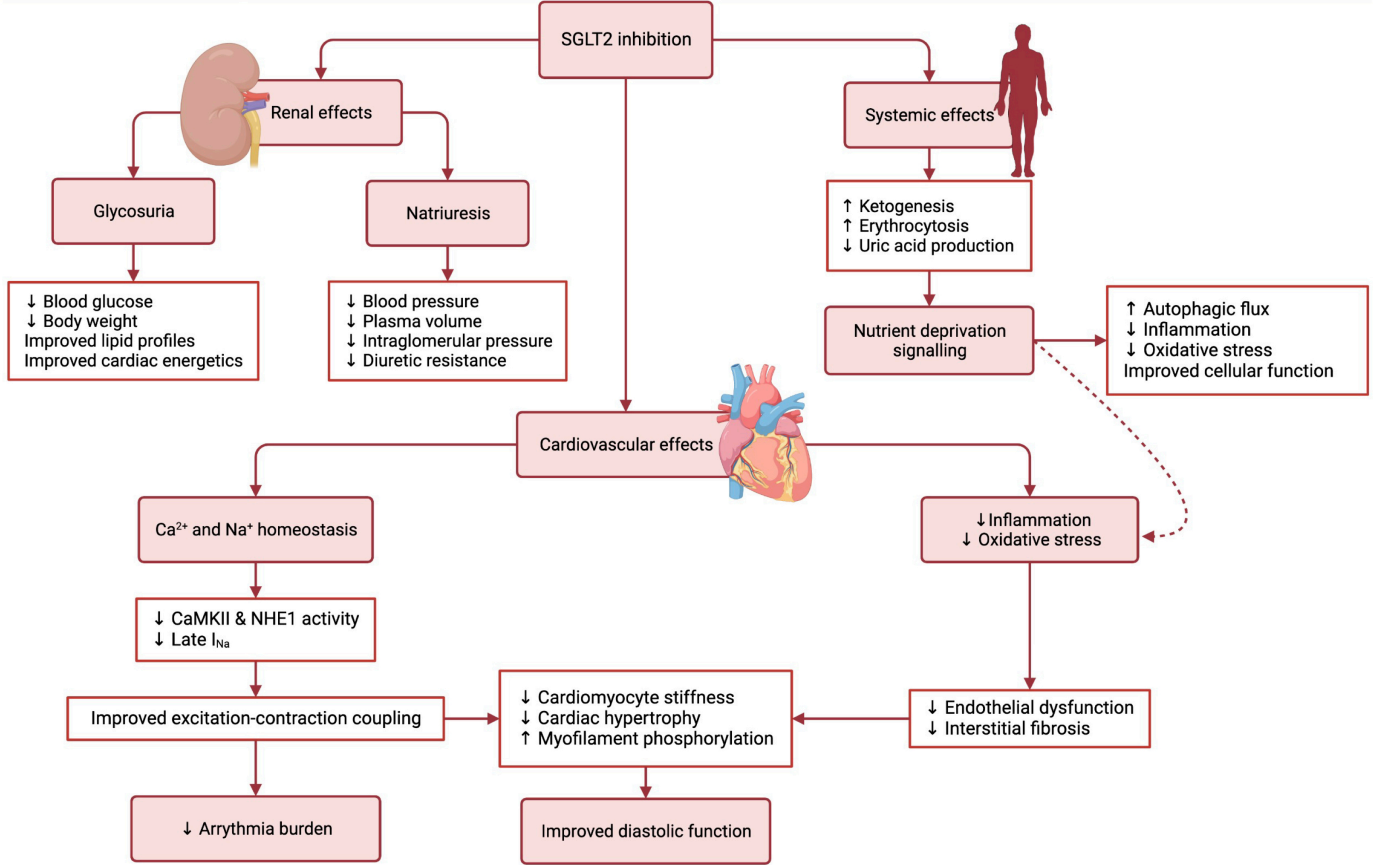
**Potential cardioprotective mechanisms of sodium-glucose cotransporter-2 inhibition in heart failure with preserved ejection fraction**. Sodium-glucose cotransporter-2 (SGLT2) inhibitors have increasingly been demonstrated to have various beneficial effects on the cardiovascular system and have recently been shown to improve outcomes in heart failure with preserved ejection fraction (HFpEF). This may be due to pleiotropic effects of SGLT2 inhibitors, well beyond their effect on glycemic control.

Fig. 1 shows the potential mechanisms of cardiovascular benefit with SGLT2 inhibitors in patients with HFpEF. In summary, the cardioprotective mechanisms behind SGLT2 inhibition in HFpEF could be related to better control of comorbidities such as diabetes mellitus, obesity and hypertension, improved mechanism of natriuresis as compared to loop diuretics, increased ketone bodies leading to more efficient energy metabolism by cardiomyocytes, reduction of cellular stress through autophagy, amelioration of endothelial function by reducing oxidative stress and systemic inflammation and cardio-specific molecular mechanisms that may improve myocardial contractility and potentially reduce the burden of arrythmias in HFpEF.

Considering that these benefits go beyond the effects of SGLT-2 inhibition, we agree that the more appropriate term to designate this new class of drugs would be gliflozins [153].

## 4. Limitations of Current Evidence

Some limitations must be considered when evaluating the evidence behind SGLT2 inhibition in HFpEF. The cardioprotective mechanisms of SGLT2 inhibition are likely pleiotropic, but are not yet fully explained. Further research is required to better understand the mechanisms behind SGLT2 inhibition.

The large randomized clinical trials studying the use of SGLT2 inhibitors in patients with HFpEF have mainly evaluated their effects on cardiovascular outcomes, and little is known about the effect of SGLT2 inhibitors on health status in these patients. Several trials are underway which may help to further understand the effect of SGLT2 inhibitors on different HFpEF phenotypes, quality of life, and exercise capacity.

Finally, it is important to note that it is frequently difficult to compare results from different trials in HFpEF due to the variability of definitions and LVEF cut-offs, with trials frequently including patients with HFmrEF (defined as an LVEF between 41–49%). It must be considered that the clinical course of patients with HFmrEF may be more similar to patients with HFrEF than with HFpEF [6]. Future trials must be cognizant of the changing definitions and classifications of patients with HF and should present results in a manner such as these patients may be more readily comparable.

## 5. Conclusions

HFpEF is an heterogenous syndrome with multiple phenotypes and several associated comorbidities, in which potential therapies must be individualized according to each patient. Among these therapies, gliflozins were the only class of drug that have been proven to change cardiovascular outcomes in HFpEF patients in a consistent and transversal manner, independent of ejection fraction, age, functional class, or diabetes status. The mechanisms behind the cardiovascular and renal benefits are multifaceted and cannot be ascribed to their effect on glycemic control.

Currently, several ongoing clinical studies are evaluating the effects of SGLT2 inhibitors on biomarkers, health status, functional status and diastolic function in patients with HFpEF, making the prospect of further understanding the mechanisms behind the cardiovascular benefit of SGLT2 inhibition an exciting time for HF research, with the potential to establish new frontiers in HFpEF management.

## Abbreviations

BNP, B-type natriuretic peptide; CaMKII, calcium/calmodulin-dependent kinase II; CI, confidence interval; CMR, cardiac magnetic resonance; eGFR, estimated glomerular filtration rate; eNOS, endothelial nitric oxide synthetase; HF, heart failure; HFmrEF, heart failure with mildly reduced ejection fraction; HFpEF, heart failure with preserved ejection fraction; HFrEF, heart failure with reduced ejection fraction; HR, hazard ratio; KCCQ, Kansas City Cardiomyopathy Questionnaire; LVEF, left ventricular ejection fraction; NHE1, sodium-hydrogen exchanger 1; NLRP3, nucleotide-binding domain-like receptor protein 3; NT-ProBNP, N-terminal pro-B type natriuretic peptide; NYHA, New York Heart Association; PKG1α, protein kinase G1 alpha; RyR2, cardiac ryanodine receptor type 2; SGLT2, sodium-glucose cotransporter-2; 6MWD, six-minute walking distance.
